# Supplementary material for: Dietary Effects of Introducing Salt-Reduced Bread with and without Dietary Counselling—A Cluster Randomized Controlled Trial
Source: Nutrients. 2022 Sep 17;14(18):3852. doi: 10.3390/nu14183852 (PMC9503308; doi:10.3390/nu14183852)
Supplement: Supplementary file 1 [file nutrients-14-03852-s001.zip › nutrients-1899270-SI.pdf]

Supplementary Table S1: Intake of food groups at baseline and estimated mean change from baseline to follow-up in the three groups (g/10MJ)

	Intervention A				Intervention B				Control			
	n*	Baseline (median [IQR])	Estimated mean change in % (95% Cl)	P-value	n*	Baseline (median [IQR])	Estimated mean change in % (95% Cl)	P-value	n*	Baseline (median [IQR])	Estimated mean change in % (95% Cl)	P-value
Children												
Food groups												
Milk and milk products	40/36	512 [338, 678]	-15 (-28, 1)	0.067	64/60	369 [261, 606]	-16 (-29, -1)	**0.043**	52/47	423 [323, 556]	-8 (-25, 12)	0.387
Cheese and cheese products	40/33	29 [17, 41]	-43 (-64, -10)	**0.015**	64/60	30 [15, 52]	-20 (-41, 8)	0.144	51/47	30 [19, 54]	8 (-21, 46)	0.634
Bread, total	40/36	155 [124, 183]	31 (7, 58)	**0.007**	64/60	170 [142, 211]	22 (8, 38)	**0.001**	52/47	178 [133, 234]	15 (2, 30)	**0.019**
Rye bread	34/33	52 [28, 96]	55 (-27, 229)	0.256	55/56	72 [31, 112]	77 (2, 206)	**0.041**	50/45	79 [40, 119]	-3 (-24, 23)	0.819
Wheat bread	40/36	93 [66, 113]	30 (3, 65)	**0.028**	64/60	97 [66, 134]	19 (-4, 48)	0.105	52/45	102 [72, 121]	4 (-25, 43)	0.821
Breakfast cereals	33/31	43 [13,75]	-19 (-57, 52)	0.503	53/52	35 [9, 99]	20 (-30, 105)	0.510	43/37	50 [12, 88]	-28 (-67, 57)	0.401
Rice and pasta	39/35	44 [23, 84]	-35 (-63, 14)	0.133	61/55	45 [28, 71]	-29 (-61, 27)	0.249	50/44	43 [19, 76]	-15 (-48, 39)	0.515
Potatoes and potato products	36/33	31 [16, 44]	73 (-28, 318)	0.219	55/49	28 [10, 52]	-19 (-64, 80)	0.606	43/35	17 [6, 34]	27 (-49, 219)	0.609
Vegetables	40/36	145 [122, 197]	5 (-16, 31)	0.701	64/60	166 [109, 227]	2 (-16, 22)	0.875	52/47	150 [94, 193]	20 (3, 39)	**0.017**
Fruit	39/36	115 [77, 165]	63 (7, 151)	**0.024**	63/60	141 [76, 182]	38 (6, 79)	**0.016**	52/47	140 [85, 174]	12 (-16, 49)	0.432
Meat and poultry	40/36	120 [93, 156]	16 (-1, 36)	0.062	64/60	119 [88, 142]	-5 (-19, 12)	0.551	52/47	104 [85, 143]	11 (-4, 30)	0.149
Fish and fish products	28/17	16 [0, 24]	-68 (-88, -18)	**0.018**	51/44	14 [3, 32]	-33 (-66, 31)	0.238	41/26	13 [4, 34]	-74 (-88, -44)	**0.001**
Processed meat, poultry and fish	40/36	51 [35, 71]	15 (-10, 48)	0.251	64/60	61 [39, 78]	7 (-11, 28)	0.502	51/46	51 [39, 89]	-1 (-19, 21)	0.910
Butter and spreads	39/36	14 [8, 22]	30 (-10, 90)	0.167	58/57	13 [7, 19]	20 (-15, 68)	0.302	47/45	24 [9, 33]	14 (-24, 72)	0.540
Salty snacks	29/23	6 [0, 12]	-24 (-66, 68)	0.491	37/35	5 [0, 18]	-21 (-64, 73)	0.561	33/33	4 [0, 14]	45 (-29, 197)	0.303
Cakes, sweets and chocolate	38/36	81 [57, 115]	80 (5, 210)	**0.032**	63/56	84 [57, 131]	-18 (-49, 31)	0.400	52/45	90 [64, 120]	-13 (-39, 22)	0.413
Sugar sweetened beverages	37/32	136 [83, 276]	16 (-42, 129)	0.672	57/55	151 [81, 285]	48 (-30, 213)	0.303	44/38	123 [52, 253]	8 (-53, 146)	0.862
Bread fillings												
Fillings, total	40/35	66 [41, 91]	-14 (-42, 27)	0.445	64/59	67 [46, 108]	-19 (-33, -2)	**0.033**	52/47	75 [54, 105]	-13 (-29, 6)	0.164
High salt (>2.5g/100g)	32/29	7 [2, 15]	8 (-50, 132)	0.847	55/49	10 [4, 24]	-28 (-58, 22)	0.222	44/41	10 [3, 18]	33 (-21, 125)	0.289
Medium salt (1-2.5g/100g)	38/31	22 [11, 46]	-18 (-60, 67)	0.589	58/53	20 [8, 36]	-2 (-40, 60)	0.928	48/43	39 [22, 57]	-34 (-58,2)	0.064
Low salt (<1.0g/100g)	38/33	28 [12, 45]	-30 (-61, 26)	0.234	62/54	29 [17, 55]	-40 (-64, 0)	0.051	49/42	24 [11, 34]	-35 (-62, 11)	0.115
Adults												
Food groups												
Milk and milk products	41/38	268 [188, 492]	-10 (-24, 6)	0.214	63/60	260 [159, 392]	-14 (-27, 1)	0.074	49/47	325 [189, 405]	-12 (-26, 6)	0.181
Cheese and cheese products	41/37	49 [33, 68]	-26 (-50, 7)	0.115	63/60	42 [27, 65]	-13 (-26, 3)	0.107	49/47	41 [19, 56]	23 (-2, 54)	0.069
Bread, total	41/38	152 [114, 199]	23 (5, 45)	**0.011**	63/60	150 [121, 186]	21 (8, 34)	**0.000**	49/47	148 [122, 168]	17 (2, 34)	**0.027**
Rye bread	40/37	70 [39, 115]	-1 (-22, 25)	0.919	60/58	73 [45, 95]	12 (-18, 54)	0.470	49/45	67 [39, 94]	-24 (-50, 15)	0.192
Wheat bread	41/38	79 [50, 108]	42 (12, 80)	**0.004**	63/60	82 [51, 109]	36 (11, 68)	**0.004**	49/47	75 [53, 114]	35 (6, 72)	**0.014**
Breakfast cereals	27/23	17 [0, 53]	-37 (-71, 38)	0.248	49/43	24 [5, 57]	-33 (-62, 19)	0.169	35/26	27 [0, 52]	-69 (-84, -38)	**0.001**
Rice and pasta	39/36	27 [17, 52]	13 (-34, 95)	0.658	60/56	40 [25, 66]	-31 (-59, 17)	0.170	47/43	41 [24, 76]	-23 (-51, 20)	0.241
Potatoes and potato products	38/34	33 [18, 64]	19 (-46, 164)	0.666	55/55	35 [16, 66]	103 (-3, 326)	0.060	45/42	38 [13, 50]	44 (-36, 225)	0.374
Vegetables	41/38	179 [119, 237]	14 (-8, 39)	0.218	63/60	247 [155, 335]	-7 (-18, 6)	0.270	49/47	229 [162, 284]	-1 (-15, 15)	0.937

Fruit	41/37	100 [44, 186]	31 (-10, 90)	0.152	62/60	102 [38, 160]	76 (26, 146)	**0.001**	49/47	97 [34, 164]	19 (-9, 55)	0.201
Meat and poultry	41/38	142 [99, 171]	21 (6, 36)	**0.003**	63/60	134 [93, 164]	3 (-10, 19)	0.685	48/46	132 [101, 157]	11 (-11, 39)	0.370
Fish and fish products	33/25	17 [4, 35]	-52 (-78, 2)	0.056	56/53	24 [7, 43]	1 (-42, 77)	0.973	42/41	21 [11, 48]	-9 (-50, 63)	0.746
Processed meat, poultry and fish	41/38	51 [33, 80]	37 (0, 88)	**0.047**	63/59	54 [37, 75]	-12 (-32, 14)	0.330	47/47	51 [32, 81]	10 (-18, 48)	0.539
Butter and spreads	35/33	12 [3, 17]	-16 (-52, 49)	0.556	59/54	10 [5, 18]	-11 (-39, 30)	0.540	47/42	18 [9, 25]	-17 (-43, 23)	0.357
Salty snacks	30/21	5 [0, 10]	-47 (-78, 23)	0.141	40/34	3 [0, 8]	-31 (-65, 36)	0.292	29/31	4 [0, 10]	24 (-40, 156)	0.566
Cakes, sweets and chocolate	40/38	87 [48, 129]	8 (-23, 51)	0.660	62/58	93 [67, 138]	-31 (-47, -10)	**0.007**	48/46	84 [64, 105]	-3 (-32, 39)	0.880
Sugar sweetened beverages	33/32	156 [41, 251]	39 (-50, 286)	0.531	52/53	106 [40, 208]	69 (-30, 310)	0.244	39/42	60 [16, 164]	171 (-0, 639)	0.051
Bread fillings												
Fillings, total	41/37	97 [62, 143]	-13 (-40, 27)	0.478	62/59	96 [66, 135]	-15 (-32, 7)	0.164	49/47	83 [63, 124]	-17 (-36, 7)	0.152
High salt (>2.5g/100g)	36/33	9 [3, 16]	21 (-37, 132)	0.574	51/48	8 [2, 19]	-20 (-55, 40)	0.437	39/39	9 [2, 16]	-4 (-53, 95)	0.905
Medium salt (1-2.5g/100g)	40/35	52 [31, 82]	-32 (-57, 9)	0.117	60/57	43 [28, 66]	-18 (-40, 12)	0.213	48/43	43 [25, 60]	-27 (-54, 15)	0.175
Low salt (<1.0g/100g)	38/36	32 [10, 46]	13 (-34, 92)	0.661	59/56	35 [14, 54]	-2 (-33, 45)	0.935	49/43	27 [16, 48]	-51 (-71, -15)	**0.011**

All analyses are based on imputed datasets and presented as mean change in % with 95% CI calculated using mixed models with age, gender, BMI, parental education, under- and acceptable energy intake as fixed effects and participant as random effect. Baseline values are based on observed values and includes participants with zero intake. All values were logtransformed before analysis. P-values in bold are significant.

*Number of participant with an intake >0g/day at baseline/follow-up

Supplementary Table S2: Estimated differences between groups at follow-up in intake of food groups (g/10MJ)

	ICC	Intervention A compared to control: Mean difference in % (95% CI)	P-value	Intervention B compared to control: Mean difference in % (95% CI)	P-value	Intervention B compared to Intervention A: Mean difference in % (95% CI)	P-value
Children							
Food groups							
Milk and milk products	0.30	7 (-19, 40)	0.633	-3 (-24, 23)	0.785	-9 (-30, 17)	0.453
Cheese and cheese products	0.46	-52 (-73, -15)	**0.011**	-23 (-54, 28)	0.316	60 (-7, 176)	0.091
Bread, total	0.39	-4 (-23, 18)	0.685	4 (-13, 24)	0.668	9 (-11, 32)	0.408
Rye bread	0.32	3 (-51, 116)	0.932	48 (-24, 186)	0.248	43 (-29, 190)	0.321
Wheat bread	0.84	9 (-33, 78)	0.732	13 (-28, 77)	0.605	3 (-36, 66)	0.892
Breakfast cereals	0.41	36 (-50, 267)	0.548	91 (-23, 370)	0.160	41 (-46, 268)	0.485
Rice and pasta	0.65	8 (-56, 164)	0.863	30 (-43, 192)	0.532	20 (-49, 182)	0.679
Potatoes and potato products	0.58	70 (-53, 512)	0.417	1 (-68, 222)	0.988	-41 (-83, 102)	0.404
Vegetables	0.59	-11 (-34, 19)	0.419	-13 (-34, 13)	0.295	-2 (-26, 30)	0.885
Fruit	0.60	32 (-14, 103)	0.205	23 (-17, 81)	0.300	-7 (-39, 41)	0.729
Meat and poultry	0.58	12 (-14, 48)	0.397	-12 (-31, 13)	0.309	-22 (-39, 1)	0.061
Fish and fish products	0.52	-10 (-73, 199)	0.860	239 (14, 908)	**0.028**	278 (18, 1109)	**0.025**
Processed meat, poultry and fish	0.41	23 (-13, 74)	0.233	11 (-19, 51)	0.511	-10 (-35, 24)	0.523
Butter and spreads	0.15	7 (-34, 71)	0.794	-18 (-46, 24)	0.345	-23 (-51, 20)	0.249
Cakes, sweets and chocolate	0.89	77 (-15, 271)	0.130	8 (-45, 111)	0.826	-39 (-70, 23)	0.169
Sugar sweetened beverages	0.43	24 (-63, 317)	0.722	77 (-41, 428)	0.308	42 (-56, 356)	0.557
Bread fillings							
Fillings, total	0.58	-6 (-40, 48)	0.803	-7 (-38, 40)	0.725	-1 (-36, 52)	0.944
High salt (>2.5g/100g)	0.29	-35 (-73, 54)	0.330	-32 (-69, 47)	0.321	4 (-55, 139)	0.929
Medium salt (1-2.5g/100g)	-	11 (-48, 139)	0.784	15 (-42, 128)	0.687	3 (-50, 116)	0.929
Low salt (<1.0g/100g)	0.25	-1 (57, 128)	0.978	12 (-47, 137)	0.774	13 (-49, 153)	0.767
Adults							
Food groups							
Milk and milk products	0.17	5 (-20, 37)	0.738	-4 (-25, 22)	0.742	-8 (-29, 18)	0.504
Cheese and cheese products	0.16	-28 (-51, 6)	0.094	-24 (-45, 7)	0.119	6 (-26, 52)	0.744
Bread, total	0.33	5 (-13, 28)	0.592	9 (-7, 29)	0.289	4 (-13, 24)	0.693
Rye bread	0.24	37 (-20, 134)	0.256	46 (-10, 136)	0.126	7 (-36, 78)	0.803
Wheat bread	0.41	-1 (-26, 34)	0.967	4 (-20, 36)	0.761	5 (-21, 40)	0.742
Breakfast cereals	0.30	94 (-32, 459)	0.218	181 (9, 629)	**0.033**	45 (-47, 297)	0.473
Rice and pasta	0.04	36 (-35, 185)	0.409	-9 (-53, 76)	0.784	-33 (-67, 35)	0.260
Potatoes and potato products	0.47	5 (-63, 194)	0.928	19 (-53, 202)	0.718	13 (-58, 204)	0.805
Vegetables	0.15	9 (-14, 37)	0.474	-5 (-22, 17)	0.638	-13 (-31, 10)	0.256
Fruit	0.13	5 (-30, 57)	0.802	43 (0, 104)	**0.049**	36 (-8, 99)	0.118
Meat and poultry	0.59	26 (1, 57)	**0.044**	4 (-15, 28)	0.676	-17 (-33, 3)	0.084
Fish and fish products	0.22	-49 (-79, 22)	0.130	17 (-46, 153)	0.700	128 (-1, 423)	0.052
Processed meat, poultry and fish	0.63	37 (-15, 119)	0.196	-10 (-41, 39)	0.646	-34 (-58, 4)	0.074
Butter and spreads	-	-30 (-63, 31)	0.263	-10 (-48, 56)	0.705	29 (-28, 132)	0.399
Cakes, sweets and chocolate	0.19	8 (-32, 72)	0.746	-24 (-50, 14)	0.188	-30 (-55, 9)	0.118

Sugar sweetened beverages	0.04	-29 (-76, 115)	0.549	-9 (-66, 142)	0.845	27 (-55, 263)	0.654
Bread fillings							
Fillings, total	0.09	6 (-30, 60)	0.788	5 (-27, 51)	0.778	0 (-33, 47)	0.981
High salt (>2.5g/100g)	0.09	43 (-36, 221)	0.379	-13 (-57, 79)	0.710	-39 (-72, 31)	0.205
Medium salt (1-2.5g/100g)	0.11	2 (-46, 91)	0.953	21 (-30, 111)	0.497	19 (-34, 116)	0.568
Low salt (<1.0g/100g)	0.14	67 (-19, 245)	0.168	67 (-12, 216)	0.114	0 (-49, 98)	0.996

Analyses are based on imputed datasets and presented as mean difference in % with 95% CI calculated using mixed models with treatment group, age, gender, BMI, parental education, and under- and acceptable reported energy intake as fixed effects and family as random effect. All values were log transformed before analysis. P-values in bold are significant. ICC = Intracluster correlation coefficient

Supplementary Table S3: Intake of energy and nutrients at baseline and estimated mean change from baseline to follow-up in the three groups

	Intervention A			Intervention B			Control		
	Baseline mean (SD)	Estimated mean change (95% Cl)	P-value	Baseline mean (SD)	Estimated mean change (95% Cl)	P-value	Baseline mean (SD)	Estimated mean change (95% Cl)	P-value
Children <18y									
n	40			64			52		
Energy (kJ/d)	7459 (2283)	-369 (-897, 158)	0.170	6827 (2382)	67 (-388, 523)	0.773	6867 (1501)	-385 (-745, -25)	**0.036**
Macronutrients									
Fat (E%)	34.6 (4.3)	0.7 (-0.6, 2.0)	0.300	33.6 (4.3)	-0.3 (-1.7, 1.0)	0.648	35.9 (4.4)	-0.2 (-1.7, 1.2)	0.744
Saturated fat (E%)	13.6 (2.3)	-0.2 (-1.0, 0.6)	0.556	12.8 (2.3)	-1.2 (-2.0, -0.4)	**0.003**	14.3 (2.8)	-0.9 (-1.8, 0.0)	0.051
Carbohydrates, total (E%)	50.2 (5.6)	-0.4 (-1.9, 1.0)	0.566	51.4 (4.9)	0.5 (-1.0, 1.9)	0.526	49.7 (4.6)	0.3 (-1.2, 1.9)	0.664
Added sugar (E%)	10.3 (4.6)	0.2 (-1.1, 1.5)	0.811	9.1 (3.6)	1.1 (0.1, 2.2)	**0.030**	9.5 (4.5)	0.7 (-0.6, 2.1)	0.291
Protein (E%)	15.3 (2.6)	-0.3 (-1.0, 0.4)	0.424	15.0 (2.5)	-0.1 (-0.6, 0.4)	0.646	14.3 (1.9)	-0.1 (-0.6, 0.5)	0.811
Dietary fiber (g/10MJ)	23 (5)	0 (-2, 1)	0.626	26 (6)	0 (-2, 1)	0.583	25 (7)	-1 (-3, -0)	**0.047**
Micronutrients									
Sodium (g/10MJ)	3.7 (0.6)	-0.5 (-0.7, -0.2)	**0.000**	4.0 (0.7)	-0.5 (-0.8, -0.2)	**0.001**	3.8 (0.6)	0.1 (-0.2, 0.3)	0.538
Potassium (g/10MJ)	3.1 (0.5)	0.1 (-0.1, 0.3)	0.255	3.2 (0.6)	0.1 (-0.1, 0.2)	0.480	2.9 (0.5)	0.1 (-0.0, 0.2)	0.112
Adults ≥18y									
n	41			63			49		
Energy (kJ/d)	9189 (2464)	-441 (-1084, 202)	0.179	8577 (2104)	587 (95, 1080)	**0.019**	9067 (1870)	-404 (-866, 58)	0.086
Macronutrients									
Fat (E%)	37.1 (5.5)	1.4 (-0.0, 2.8)	0.058	36.8 (4.0)	0.3 (-0.9, 1.4)	0.663	37.9 (5.4)	0.9 (-0.5, 2.3)	0.208
Saturated fat (E%)	14.0 (2.7)	0.2 (-0.4, 0.8)	0.580	13.6 (2.4)	-0.5 (-1.2, 0.2)	0.133	14.2 (2.9)	-0.1 (-0.9, 0.7)	0.891
Carbohydrates, total (E%)	46.8 (5.9)	-1.7 (-3.3, -0.0)	**0.048**	46.8 (4.3)	-0.3 (-1.6, 1.1)	0.707	46.3 (5.8)	-0.8 (-2.3, 0.7)	0.294
Added sugar (E%)	9.9 (6.7)	-0.6 (-2.0, 0.8)	0.382	8.3 (4.0)	0.4 (-0.6, 1.4)	0.452	8.7 (5.3)	0.7 (-0.4, 1.8)	0.236
Protein (E%)	16.1 (2.4)	0.2 (-0.6, 1.1)	0.567	16.4 (2.3)	0.0 (-0.6, 0.6)	0.999	15.9 (2.5)	0.0 (-0.7, 0.6)	0.894
Dietary fiber (g/10MJ)	24 (7)	-1 (-3, 1)	0.195	26 (6)	-2 (-3, -0)	**0.020**	25 (6)	-2 (-4, -1)	**0.007**
Micronutrients									
Sodium (g/10MJ)	3.9 (0.7)	-0.2 (-0.5, 0.0)	0.064	4.1 (0.8)	-0.5 (-0.7, -0.3)	**0.000**	4.1 (0.7)	-0.1 (-0.3, 0.2)	0.570
Potassium (g/10MJ)	3.4 (0.6)	0.1 (-0.1, 0.3)	0.189	3.6 (0.6)	0.1 (-0.1, 0.2)	0.490	3.5 (0.6)	0.0 (-0.2, 0.2)	0.936

All analyses are based on imputed datasets and presented as mean change with 95% CI calculated using mixed models with age, gender, BMI, parental education, and under- and acceptable reported energy intake as fixed effects and participant as random effect. P-values in bold are significant.

Supplementary Table S4: Estimated differences between groups at follow-up in intake of energy and nutrients (g/10MJ)

		Intervention A compared to control		Intervention B compared to control		Intervention B compared to Intervention A	
	ICC	Mean difference (95% CI)	P-value	Mean difference (95% CI)	P-value	Mean difference (95% CI)	P-value
Children <18y							
Energy (kJ/d)	0.39	49 (-478, 575)	0.856	219.9 (-260.6, 700.3)	0.370	171 (-334, 676)	0.506
Macronutrients							
Fat (E%)	0.47	0.0 (-2.3, 2.3)	0.990	-1.4 (-3.5, 0.7)	0.197	-1.4 (-3.6, 0.8)	0.214
Saturated fat (E%)	0.53	0.0 (-1.3, 1.4)	0.977	-1.3 (-2.5, -0.1)	**0.040**	-1.3 (-2.6, -0.0)	**0.046**
Carbohydrates, total (E%)	0.42	-0.3 (-2.7, 2.2)	0.839	1.2 (-0.9, 3.4)	0.269	1.5 (-0.8, 3.8)	0.213
Added sugar (E%)	0.58	0.1 (-2.0, 2.3)	0.897	0.6 (-1.4, 2.5)	0.557	0.4 (-1.6, 2.5)	0.678
Protein (E%)	0.31	0.2 (-0.7, 1.0)	0.678	0.2 (-0.6, 0.9)	0.672	0.0 (-0.8, 0.8)	0.975
Dietary fiber (g/10MJ)	0.41	0 (-2, 2)	0.960	1 (-1, 3)	0.215	1 (-1, 3)	0.267
Micronutrients							
Sodium (g/10MJ)	0.67	-0.6 (-1.0, -0.1)	**0.015**	-0.4 (-0.8, 0.0)	0.061	0.2 (-0.3, 0.6)	0.437
Potassium (g/10MJ)	0.50	0.1 (-0.2, 0.3)	0.588	0.0 (-0.2, 0.3)	0.803	0.0 (-0.3, 0.2)	0.744
Adults ≥18y							
Energy (kJ/d)	0.16	-78 (-676, 520)	0.799	841 (311, 1372)	**0.002**	919 (345, 1493)	**0.002**
Macronutrients							
Fat (E%)	0.20	-0.1 (-1.9, 1.8)	0.946	-1.3 (-2.9, 0.4)	0.132	-1.2 (-3.0, 0.6)	0.181
Saturated fat (E%)	0.24	0.1 (-1.0, 1.1)	0.909	-0.7 (-1.7, 0.2)	0.117	-0.8 (-1.8, 0.2)	0.113
Carbohydrates, total (E%)	0.25	-0.5 (-2.7, 1.8)	0.674	1.0 (-1.0, 3.0)	0.325	1.5 (-0.7, 3.6)	0.173
Added sugar (E%)	0.42	-1.3 (-3.2, 0.6)	0.178	-0.5 (-2.2, 1.2)	0.564	0.8 (-1.0, 2.6)	0.386
Protein (E%)	0.36	0.6 (-0.5, 1.6)	0.306	0.3 (-0.7, 1.2)	0.559	-0.3 (-1.3, 0.7)	0.601
Dietary fiber (g/10MJ)	0.37	1 (-1, 3)	0.493	1 (-1, 3)	0.267	0 (-2, 2)	0.748
Micronutrients							
Sodium (g/10MJ)	0.48	-0.3 (-0.6, 0.1)	0.115	-0.4 (-0.7, -0.1)	**0.008**	-0.1 (-0.5, 0.2)	0.408
Potassium (g/10MJ)	0.20	0.2 (-0.1, 0.4)	0.277	0.1 (-0.2, 0.3)	0.597	-0.1 (-0.4, 0.2)	0.515

Analyses are based on imputed datasets and presented as mean difference with 95% CI calculated using mixed models with treatment group, age, gender, BMI, parental education, and under- and acceptable reported energy intake as fixed effects and family as random effect. P-values in bold are significant. ICC = Intracluster correlation coefficient
